# Comparing Myelosuppression Frequency in Indian Inflammatory Bowel Disease Patients: A Randomized Trial of Full Dose Versus Gradual Escalation of Thiopurines

**DOI:** 10.7759/cureus.50969

**Published:** 2023-12-22

**Authors:** Alok Kumar Singh, Sanjeev Sachdeva, Siddharth Srivastava, Ujjwal Sonika, Ajay Kumar, Barjesh C Sharma, Amarender S Puri, Ashok Dalal

**Affiliations:** 1 Department of Gastroenterology, G.B. Pant Hospital, New Delhi, IND; 2 Department of Gastroenterology, Medanta - The Medicity Hospital, Gurugram, IND

**Keywords:** inflammatory bowel disease, myelosuppression, ulcerative colitis, leukopenia, azathioprine

## Abstract

Introduction: We aimed to compare the frequency of myelosuppression in patients initiating azathioprine (AZA) at full dose versus those undergoing gradual dose escalation.

Methods: Forty patients with inflammatory bowel disease were recruited over one year and randomized into two groups of 20. Group A initiated AZA at a full dose of 2 mg/kg, while group B started at 1 mg/kg with subsequent dose increases at regular intervals.

Results: Seventeen patients from each group were included in the final analysis. During follow-up, two patients (11.8%) from group A and four patients (23.5%) from group B experienced relapses (p=0.65). Myelosuppression occurred in two patients (11.8%) from each group. Absolute neutrophil counts in group A tended to have lower median values than those in group B, particularly four weeks after AZA initiation. Univariate analysis identified serum proteins, albumin, and bilirubin as significantly associated with leukopenia, but these factors were not significant according to multivariate analysis.

Conclusions: The incidence of myelosuppression was similar between the groups. Patients with full-dose initiation of AZA had numerically fewer relapses during the follow-up period.

## Introduction

Thiopurines, namely 6-mercaptopurine and azathioprine (AZA), are the cornerstones of maintenance therapy for ulcerative colitis (UC) and Crohn’s disease (CD) [1,2]. A study conducted in India reported the use of 6-mercaptopurine or AZA in 30% of UC patients and 63% of CD patients [3]. Despite their efficacy, thiopurines are associated with a range of adverse events, including gastrointestinal intolerance, myelosuppression, pancreatitis, hepatitis, infections, and malignancies. Alarmingly, up to one-quarter of patients with inflammatory bowel disease (IBD) discontinue thiopurine treatment within the initial months due to these adverse events [4-6]. Myelosuppression stands out as the most critical and potentially fatal reaction to AZA, with a 7% cumulative incidence of myelotoxicity, primarily manifesting in the initial weeks of therapy [7].

We hypothesized that a gradual increase in the AZA dose might offer a safer treatment strategy for patients with IBD, allowing for improved tolerability and early detection of myelosuppression. Our main goal was to assess the frequency of myelosuppression in patients beginning with a full dose of AZA compared to those treated with a stepwise dose increase. Our secondary objectives were to investigate other AZA-related adverse events, identify predictors, and monitor the frequency of relapses during the follow-up period in UC patients. To our knowledge, this was the first study of its kind.

## Materials and methods

Study design

This was an open-label randomized controlled trial conducted at the Department of Gastroenterology, G.B. Pant Institute of Postgraduate Medical Education and Research (GIPMER), over 12 months from May 2020 to May 2021. The Institutional Ethics Committee of Maulana Azad Medical College, New Delhi, India and associated hospitals approved the study (approval letter no. F.1/IEC/MAMC/72/07/2020/No. 82, dated May 15, 2020). Prior to participation, written and informed consent was obtained from all individuals enrolled in the study.

Sampling and randomization

During the designated period, we aimed to recruit 50 patients diagnosed with IBD. However, the COVID-19 pandemic impacted the recruitment process, resulting in a final cohort of 40 patients. These individuals, either admitted to the gastroenterology department wards or attending the gastroenterology outpatient department (OPD) IBD clinics at GIPMER, were evenly divided into two groups, A and B, each comprising 20 patients. The randomization was executed in a 1:1 ratio using computer-generated tables.

Given the lack of preceding studies of a similar nature, the sample size was determined using a convenience method. This approach was informed by the number of IBD patients requiring initiation of AZA therapy and encountered in the gastroenterology OPD or ward. The inclusion of 20 patients per group was based on the IBD patient data collected by the gastroenterology department over the preceding three years.

Patient selection

The study enrolled consecutive IBD patients who were indicated for AZA therapy and encountered at the gastroenterology OPD or wards. The inclusion criteria were broad to capture a representative sample of individuals requiring AZA treatment. Moreover, exclusion criteria were applied rigorously, as follows: patients younger than 18 or older than 70 years; those with active tuberculosis; hemoglobin levels below 8 g/dL; total leucocyte count (TLC) under 4000 cells/mm^3^; platelet count below 100,000/mm^3^; liver enzymes (aspartate aminotransferase or alanine aminotransferase) exceeding twice the upper limit of normal; active infections or fever; current or past lymphoma; pregnancy; previous pancreatitis; any form of acute or chronic kidney disease; or positive status for hepatitis B, hepatitis C, or HIV. The diagnosis of IBD, whether UC or CD, was established based on clinical presentation and corroborated by suitable radiological, endoscopic, and histopathologic evidence.

Treatment protocol

Group A commenced with a full dose of AZA at 2 mg/kg, while group B (the gradual escalation group) started with 1 mg/kg, with doses increasing to 1.5 mg/kg and 2 mg/kg after four and eight weeks, respectively. The criteria for initiating AZA therapy were steroid-dependent or steroid-refractory disease and frequent relapses or as part of combination therapy for treatment-naïve patients presenting with severe disease. The definitions for “steroid-dependent,” “steroid-refractory,” and “frequent relapses” adhered to the standards set by the European Crohn’s and Colitis Organisation [8].

Initial assessments and monitoring

Prior to initiating therapy, baseline liver function tests (LFTs) and complete blood counts were performed. Additional screening tests, including hepatitis B surface antigen, IgM anti-hepatitis C virus, HIV testing, and a chest X-ray, were also conducted. Scheduled laboratory monitoring of complete blood counts and LFTs was recommended at 2, 4, 6, 8, 12, 20, and 24 weeks following the start of treatment. All participants were clinically monitored for 24 weeks after commencing treatment. If investigations revealed any abnormalities, adjustments to the dosage or discontinuation of treatment were made, depending on the severity of the values and side effects observed. Hepatic dysfunction was characterized by an increase in alanine aminotransferase levels to more than twice the upper limit of normal. Leukopenia was defined as leukocyte counts below 3000 cells/mm^3^, with severe leukopenia noted as counts under 1000 cells/mm^3^. Thrombocytopenia was identified when platelet counts fell below 100,000 cells/mm^3^, with severe thrombocytopenia at counts under 20,000 cells/mm^3^ [9,10]. Severe anemia was defined as blood hemoglobin levels less than 8 g/dL. Patients were advised to promptly report any side effects, including gastrointestinal intolerance, abdominal pain (suggestive of pancreatitis), the presence of any lump (suggestive of lymphoma), unexpected bleeding or bruising, symptoms of anemia, or fever.

Statistical analysis

Data were analyzed with IBM SPSS Statistics for Windows, Version 25 (Released 2017; IBM Corp., Armonk, New York, United States). Medians with ranges, along with proportions, were used to evaluate continuous and categorical data, respectively. The Mann-Whitney U test was used for the analysis of quantitative data, while Fisher's exact test was used for qualitative data. P-values less than 0.05 were considered statistically significant.

## Results

**Figure 1:**
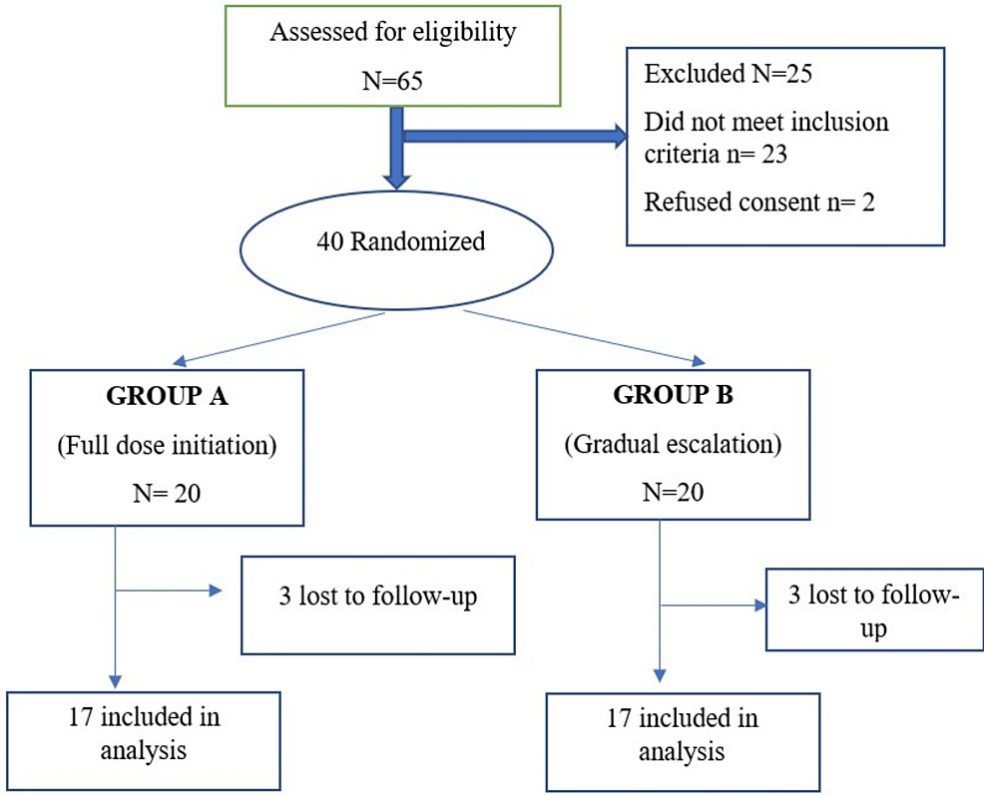
Consort diagram

A total of 65 patients were assessed for eligibility, among whom 25 were excluded, leaving 40 patients who were randomized into two groups in a 1:1 ratio using computer-generated tables. Each group lost three patients to follow-up. Consequently, 17 patients from each group were included in the final analysis, as depicted in Figure 1.

In group A, 35.3% of the patients were men, compared with 47.1% in group B. The median ages were 32 years in group A and 30 years in group B. The diagnosis of UC was present in 14 patients from group A and 15 from group B. The CD was diagnosed in three patients from group A and two from group B. All patients with CD presented with ileocolonic disease and stricturing, as revealed by imaging studies. Additionally, two CD patients in each group had perianal disease (Table 1).

**Table 1:** Baseline characteristics of study subjects UC: Ulcerative colitis

	Group A (n=17)	Group B (n=17)	p-value
Gender			
Male	6 (35.3%)	8 (47.1%)	0.72
Female	11 (64.7%)	9 (52.9%)	
Median age in years	32 (21-50)	30 (19-45)	0.18
Diagnosis			
Ulcerative colitis	14 (82.4%)	15 (88.2%)	1.0
Crohn’s disease	3 (17.6%)	2 (11.8%)	
Extent of UC			
(till splenic flexure) E2	1 (7.1%)	3 (20.0%)	0.59
(proximal to splenic flexure) E3	13 (92.9%)	12 (80.0%)	
Extraintestinal manifestations (EIM)			
No	13 (76.5%)	14 (82.4%)	1.0
Yes	4 (23.5%)	3 (17.6%)	
Indication for starting azathioprine			
Frequent relapse	4 (23.5%)	6 (35.3%)	0.86
Steroid dependent UC	6 (35.3%)	6 (35.3%)	
Steroid resistant UC	4 (23.5%)	3 (17.6%)	
Crohn’s disease	3 (17.6%)	2 (11.8%)	
Infliximab (IFX)			
No	13 (76.5%	12 (70.6%)	1.0
Yes	4 (23.5%)	5 (29.4%)	

The absolute neutrophil counts (ANC) in group A exhibited a trend toward lower median values compared with those in group B, especially four weeks after AZA, with p-values reaching statistical significance at 6, 8, and 20 weeks after AZA initiation (Table 2).

**Table 2:** ANC in study subjects at different follow-ups: median (range) ANC: Absolute neutrophil count

ANC count (cells/mm^3^)	Group A	Group B	p-value
Baseline	3600 (2500-5500)	3400 (2780-5600)	0.80
2 weeks	2900 (1980-4080)	3600 (2160-5600)	0.09
4 weeks	2700 (90-6000)	3600 (2400-6000)	0.05
6 weeks	2725 (1900-3800)	3600 (2300-4800)	<0.01
8 weeks	2740 (1100-3460)	3800 (2670-4800)	<0.01
12 weeks	3100 (2000-4090)	3350 (1300-4790)	0.07
20 weeks	3350 (1860-3980)	3800 (2700-4500)	0.02
24 weeks	3400 (2540-4060)	3870 (1290-4120)	0.29

TLCs were similar between the groups throughout the follow-up period (Table 3).

**Table 3:** TLC in study subjects at different follow-ups: median (range) TLC: Total leucocyte count

TLC count (cells/mm^3^)	Group A (n=17)	Group B (n=17)	p-value
Baseline	6300 (5000-10500)	7500 (5700-11000)	0.35
2 weeks	6500 (4200-8200)	7500 (4200-10000)	0.34
4 weeks	6400 (300-11000)	7000 (4300-11000)	0.53
6 weeks	5600 (4200-8000)	6700 (4400-9000)	0.15
8 weeks	5850 (2200-8000)	6600 (4800-9000)	0.13
12 weeks	6500 (4200-8200)	6500 (2200-9000)	0.77
20 weeks	6900 (4100-8200)	7200 (5000-8200)	0.36
24 weeks	7000 (5000-8400)	7600 (2267-7850)	0.85

Myelosuppression developed in two patients (11.8%) from each group (p>0.99), with three of the four patients experiencing reversal of myelosuppression upon cessation of AZA. AZA was subsequently reintroduced in these three patients. One patient in group A experienced severe myelosuppression after four weeks of AZA therapy, presenting with a TLC of 300 cells/mm^3^ and an ANC of 90 cells/mm^3^, four weeks after AZA initiation. This patient ultimately died from infections at a tertiary care hospital. Investigations for other potential causes were inconclusive, thus the pancytopenia was attributed to AZA. The median time to the development of leukopenia was 10 weeks (range 4-12 weeks). Table 4 presents the frequency of adverse events.

**Table 4:** Other adverse effects on study subjects GI: Gastrointestinal

Other adverse effects	Group A (n=17)	Group B (n=17)	p-value
Anemia	3 (17.6%)	2 (11.8%)	1.0
Thrombocytopenia	1 (5.9%)	0	1.0
GI upset	8 (47.1%)	5 (29.4%)	0.48
Pancreatitis	0	1 (5.9%)	1.0

All cases of anemia were characterized as microcytic and hypochromic, stemming from iron deficiency. Iron supplementation successfully resolved the anemia in all but one patient. The individual who developed thrombocytopenia was the same patient from group A who experienced severe pancytopenia. In group B, one patient exhibited symptoms of mild pancreatitis, including abdominal pain, which resolved within one week. AZA therapy was not initiated for this patient. The predominant gastrointestinal symptoms reported were nausea, bloating, and a sensation of abdominal fullness. Notably, no patients exhibited jaundice or abnormal LFT results.

During the observation period following the initiation of AZA treatment, two patients (11.8%) in group A and four patients (23.5%) in group B experienced relapses (p=0.65). Univariate analysis revealed that baseline serum protein, albumin, and bilirubin levels were significantly associated with the development of leukopenia (Table 5). However, these factors did not retain their statistical significance in the multivariate analysis.

**Table 5:** Association of different factors with leukopenia: median (range) TLC: Total leucocyte count; ANC: Absolute neutrophil count; AST: Aspartate aminotransferase; ALT: Alanine aminotransferase; IBD: Inflammatory bowel disease; EIM: Extraintestinal manifestations; IFX: Infliximab; UC: Ulcerative colitis

	Leukopenia absent (n=30)	Leukopenia present (n=4)	p-value
Median age (years)	31.5 (19-50)	28.5 (24-35)	0.52
Gender (male)	11	3	0.28
Baseline serum protein (gm/dl)	6.5 (5.8-7.2)	5.5 (5-6.8)	0.04
Baseline serum albumin (gm/dl)	3.75 (2-4.5)	2.6 (1.9-3.7)	0.01
Baseline Hemoglobin (gm/dl)	10.6 (8.5-12.6)	9.9 (9-10)	0.20
Baseline TLC (cells/mm^3^)	6650 (5000-11000)	8200 (6000-10500)	0.11
ANC (cells/mm^3^)	3600 (2500-5600)	3650 (3080-5500)	0.57
Bilirubin (mg/dl)	0.85 (0.2-1.1)	0.45 (0.2-0.5)	0.01
AST (U/L)	22 (11-32)	22 (19-27)	0.38
ALT (U/L)	26 (16-36)	28 (9-28)	0.56
Platelet count (cells/mm^3^)	3.6 (1.5-5.0)	2.95 (2.1-4.0)	0.37
Age (<40 years)	25	4	1.0
Type of IBD (UC)	26	3	0.48
Weight (kg)	48 (44-56.5)	42.5 (40-51.75)	0.27
Duration of disease (years)	4 (2-6.5)	1.5 (0.62-4.25)	0.08
EIM	6	1	1.0
Use of IFX	8	1	1.0

## Discussion

This study was designed to evaluate the safety and efficacy of two AZA dosing strategies: full dose initiation versus gradual escalation. Myelosuppression developed in two patients (11.8%) within each group, indicating that the incidence was comparable between the two strategies. Consequently, initiating treatment with a full dose did not increase the risk of myelosuppression. This parallels findings from a review of thiopurine-induced myelotoxicity in patients with IBD, which encompassed 66 trials with 8302 patients [7], reporting a 7% cumulative incidence of myelotoxicity-aligning with our results-predominantly manifesting in the early stages of treatment. In our study, the ANCs in group A trended toward lower median values relative to those in group B, especially four weeks after starting AZA. The cumulative incidence of myelotoxicity was similar between studies investigating standard doses (AZA at 2.0-2.5 mg/kg or mercaptopurine at 1.0-1.5 mg/kg) and those administering lower doses (AZA < 2 mg/kg or mercaptopurine < 1 mg/kg), with incidences of 6.3% and 7.1%, respectively [7]. These findings are congruent with our study, which also observed no significant difference in the incidence of myelosuppression between the groups. Another study [11] involving 695 patients found that 45 (6.5%) developed leukopenia within a median timeframe of 56 days (range 29-112 days). In a Dutch study with 363 patients over 8 years, myelosuppression occurred in 12% of patients [5]. In research from India, leukopenia developed in 13 of 111 patients (11.8%) [12]. Despite this, AZA was generally well-tolerated, with only 16.22% of patients experiencing side effects necessitating drug discontinuation. In our study, gastrointestinal side effects were more prevalent in group A (47.1%) than in group B (29.4%), although this difference was not statistically significant (p=0.48). Gastrointestinal intolerance did not necessitate any dose modifications or discontinuation of therapy for patients in either group. Anemia was observed in three patients (17.6%) from group A and two patients (11.8%) from group B, while pancreatitis was reported in only one patient from group B. Comparative data from a study by Sood et al. with 255 patients showed that 74 (29.0%) patients experienced adverse events leading to the discontinuation of AZA, with 46 (18.0%) of these cases occurring within the first four months of therapy [13]. The most frequent adverse events in that study were myelotoxicity (7.1%), hepatotoxicity (5.5%), flu-like symptoms (5.1%), and gastrointestinal issues, mainly nausea or vomiting (4.7%), along with three instances of nonmelanoma skin cancer (1.2%). Another study by Chaparro et al. [6] documented a cumulative incidence of adverse events of 26%, with an annual risk of 7% per patient year. The most frequently reported side effects were nausea (8%), myelotoxicity (4%), hepatotoxicity (4%), and pancreatitis (4%). Therefore, while the incidence of gastrointestinal side effects in our study appeared higher than the rates determined by these studies, the clinical impact was minimal. AZA was generally well-tolerated upon continued use, and gastrointestinal side effects were not significant enough to warrant discontinuation.

During the post-initiation observation period of AZA treatment, two patients (14.3%) in group A and four patients (26.7%) in group B experienced relapses (p=0.65). A study [1] revealed that the odds ratio for AZA in maintaining remission increased from 1.20 at 1 mg/kg to 4.13 (95% CI, 1.59-10.71) at 2.5 mg/kg, underscoring the significance of proper dosing. Further research [14] found that 15 of 130 patients (11.5%) who had been on thiopurines for over four years relapsed, with a higher frequency associated with CD than with UC. Although our study indicated that the full dose initiation group had fewer relapses than the gradual escalation group, this did not reach statistical significance, possibly due to type II (β) error. Consequently, larger studies are warranted to draw definitive conclusions.

Baseline serum protein, albumin, and bilirubin levels were initially associated with leukopenia in our univariate analysis. However, these associations were not confirmed in the multivariate analysis. A study by Qiu et al. [15] identified lower baseline hemoglobin and concurrent use of 5-aminosalicylic acid as independent predictors of leukopenia. Moreover, the risk of myelotoxicity was found to be significantly higher among patients treated with mercaptopurine and in female patients in another study [6].

Our study had several limitations. The sample size was small, and the research was conducted at a single center. There was no analysis of thiopurine methyltransferase or nudix hydrolase-15 mutations, nor were metabolite levels measured for monitoring. Additionally, the study population was restricted to adults and the follow-up period after AZA initiation was limited to six months.

## Conclusions

This study concluded that patients who commenced treatment with a full dose of AZA experienced fewer relapses during follow-up compared with those in the gradual escalation group. While these findings were not statistically significant, the possibility of a type II (β) error owing to the small sample size must be considered. Subsequent studies with larger sample sizes are needed to potentially confirm the findings of this study with statistical significance. The side effects observed were similar between the groups. Notably, patients with lower baseline serum protein, albumin, and bilirubin levels might be predisposed to an increased risk of developing leukopenia.
